# Virtual Ultrasound-Guided Central Vascular Access Using Apple Vision Pro: An Evaluation and Proof of Concept

**DOI:** 10.7759/cureus.87274

**Published:** 2025-07-04

**Authors:** Tobias Bexten, Adrian Boehm, Jack Miller, Suraj M Yalamuri, Matthew D Read, Trevor Simard, Stefan Wiebe

**Affiliations:** 1 Intensive Care Unit, Helios Dr. Horst Schmidt Clinic, Wiesbaden, DEU; 2 Anesthesiology and Critical Care, Mayo Clinic, Rochester, USA

**Keywords:** apple vision pro, central venous catheter (cvc), medical simulation, mixed reality in medicine, procedural training, ultrasound guidance, virtual reality (vr)

## Abstract

Background: Central venous catheter (CVC) insertion is a frequently performed procedure that requires precise hand-eye coordination and image interpretation. Despite ultrasound guidance being the standard of care, some problems, especially in ergonomics, like misalignment between the ultrasound monitors and the puncture site, remain a challenge. This study aimed to assess the integration of real-time ultrasound imaging into the Apple Vision Pro (AVP) headset (Apple Inc., Cupertino, CA) for ultrasound-guided CVC cannulation in a simulated environment. Specifically, we aimed to (1) evaluate technical feasibility, (2) quantify usability using an adjusted system usability questionnaire, and (3) compare usability perceptions across different experience levels.

Methods: In a prospective, observational pilot study, 42 intensivists performed CVC cannulation on a mannequin using a wireless ultrasound probe (GE Vscan Air™ CL, GE Healthcare, Chicago, IL) and AVP. Participants were grouped according to their experience and assessed through an evaluation that included a usability questionnaire, an observer checklist, and qualitative interviews. Usability, image quality, subjective satisfaction, and task performance were evaluated.

Results: Both junior and senior participants gave high ratings for the system’s usability, image clarity, and educational potential, with no significant differences between groups. Notable strengths included intuitive handling, improved ergonomics, and precise image placement. The challenges identified were image resolution in the near field, as well as depth perception. Qualitative feedback supported the innovational character of the system and its potential sterile workflow.

Conclusion: Integrating real-time ultrasound guidance into the AVP is feasible and highly rated by users with varying levels of experience. This concept promises to improve ergonomics, the CVC accuracy, and medical education. Further studies in clinical environments are needed to validate our findings and the impact on patient outcomes.

## Introduction

Central venous catheter (CVC) insertion is a common procedure in clinical practice, performed alone in the USA more than 5 million times annually [1]. Despite its routine use, complication rates remain as high as 15% [2]. Accurate placement of the catheter is essential to minimize risks, and ultrasound guidance has become the standard of care to improve precision [3]. However, mastering this technique requires substantial training, particularly in identifying anatomical landmarks and maintaining needle alignment. A persistent challenge is the misalignment between the visual representation of anatomical structures and their actual physical location [4]. The ultrasound monitor is often located outside direct sight, negatively impacting hand-eye coordination. Consequently, this can negatively affect ergonomics, work efficiency, and patients’ safety [5]. We hypothesized that integrating real-time ultrasound imaging into a mixed-reality headset would improve procedural accuracy and user experience in simulated CVC placement.

Augmented reality (AR) ultrasound guidance with head-mounted displays (HMDs) has been reported to achieve very high first-attempt success without access-related complications [6]. Additionally, it has been proven to reduce head movement and thus reduce interruption in the cannulation process [7]. In recent studies, optical see-through AR devices have been considered a new approach for improving usability [8]. Nevertheless, these displays are very sensitive to light conditions and have been reported to have inadequate resolution and a non-adjustable field of view.

Recent advancements in virtual reality (VR) technology, particularly the development of high-resolution, immersive systems such as the Apple Vision Pro (AVP; Apple Inc., Cupertino, CA), offer a potential alternative. The AVP integrates real-time imaging with enhanced visualization, making it a suitable tool for medical applications, including procedural training and anatomical visualization [9-11]. As such, it has been shown to be efficient in perioperative planning and performance [12,13].

The objective of this study was to assess the integration of real-time ultrasound imaging into the AVP headset for ultrasound-guided CVC cannulation in a simulated environment. Specifically, we aimed to (1) evaluate technical feasibility, (2) quantify usability using an adjusted system usability questionnaire, and (3) compare usability perceptions across different experience levels.

By integrating real-time ultrasound imaging with immersive VR, the system aims to increase procedural accuracy. The study will assess user experience, task performance, and the potential of this approach to further integration in patient care, comparing staff in the ICU based on their experience level.

## Materials and methods

The study was designed as a prospective, exploratory, nonrandomized observational study.

The objective was to assess the integration of real-time ultrasound imaging into the AVP headset for ultrasound-guided CVC cannulation in a simulated environment. Specifically, we aimed to (1) evaluate technical feasibility, (2) quantify using an adjusted system usability questionnaire, and (3) compare usability perceptions across different experience levels.

Study setting

The study was conducted in a simulated intensive care unit environment using a soft tissue training system for teaching internal jugular vascular access methods.

All participants were informed in advance about the study’s purpose, procedures, and conditions. Participation was voluntary, data collection was anonymous, and consent for scientific use of aggregated data was implied by participation.

Participants were recruited voluntarily from the Third International Extracorporeal Membrane Oxygenation (ECMO) Symposium (Queenstown, November 2024) and the Intensive Care Department and Anaesthesiology Department of Helios Dr. Horst Schmidt Hospital, Wiesbaden, Germany.

Technical aspects

GE Vscan Air™ CL (GE Healthcare, Chicago, IL) served as the ultrasound probe. As software, the GE Vscan APP (Version 3.0.1.19887) was used. The AVP functioned as VR glasses. This offers a resolution of 3660 x 3200 pixels per eye and depth of contrast far above the recommended ≥ 250/1 and a brightness of 5000 NITS (10 times the recommended for medical imaging). The 90 Hz refresh rate exceeds the recommended 60 Hz, with a response time of 12 ms. With respect to gray scaling, the AVP allows 1024 gray levels (10 bits per channel), which is significantly higher than the recommended 256 gray levels (8 bits per channel) [14].

As a mannequin, we used the Blue Phantom™ Ultrasound Central Line Training Model - Head and Torso (TruUltra), manufactured by CAE Healthcare (Sarasota, FL). The vessel depth and anatomy were fixed and could not be altered during the procedure.

Study procedure

All participants received a brief introduction and demonstration of the ultrasound probe and the AVP. For this, a five-minute explanation of the AVP setup and connection to the probe was provided, along with assistance as needed. The individual eye-hand calibration on the AVP was performed under Apple's official instructions.

Evaluation

A standardized questionnaire based on the System Usability Scale (SUS) is used as a survey instrument to evaluate participants’ self-perceived usability of the system, as described in the Appendix. The questionnaires contain questions on the following aspects: (a) user-friendliness of the ultrasound probe in the virtual environment, (b) image quality in the VR environment, (c) subjective satisfaction and future implications, and (d) perceived efficiency and effectiveness of the probe.

Simultaneously, an observer evaluated performance using a standardized checklist and conducted post-task interviews, as described in the Appendix. There was one individual observer present in all observations, accompanied by a second observer who confirmed the written comments after the intervention. The observer was not blinded to the experience level. The procedure was segmented into three phases: (a) setup and preparation, (b) venous puncture, and (c) guidewire handling. Puncture success was confirmed by ultrasound imaging.

The closing interview contained favorable and critical feedback and recommendations for future applications and adoption of the procedure. Qualitative analysis was performed based on Mayring, a systematic approach to interpret data by formulating categories and sub-summarizing patterns and statements within the interviews and open questions [15].

Statistics and data preparation

All paper-based questionnaires were transferred to an Excel sheet (Microsoft Corporation, Redmond, WA) following the two-person rule. The questionnaire involves mainly ordinal data, and open-ended questions account for nominal data. The answers were recorded on a five-point Likert scale (from 1 = strongly disagree to 5 = strongly agree) and a semantic seven-point Likert scale (e.g., hindering - supportive).

The bar charts display the mean values. Due to the restricted range of the Likert scale, the median offered limited visual differentiation in the figures. For statistical testing, depending on the type and distribution of the data, analyses were conducted using the Mann-Whitney U test based on median values, with a significance level of α = 0.05. In the text, results are reported as medians (IQRs).

Work experience was dichotomized as less than three years (junior experience) or more than or equal to three years (senior experience) for further evaluation.

Accordingly, depending on the data type and distribution, the Mann-Whitney U test was used at a significance level of alpha 5%. Moreover, depending on the distribution, the data are presented as medians (IQRs); for better visualization, the graphs show the mean values.

## Results

Baseline

In total, 42 intensivists with no dropouts participated in the survey. Seventeen (40.5%) participants had less than three years of experience (junior), and 25 (59.5%) had at least three years of experience (senior). The self-perceived median competence level was 4 (IQR: 2) among juniors and 5 (IQR: 0) among seniors on a five-point Likert scale. This difference was statistically significant (p = 0.003), with a rank-biserial correlation of r = -0.487 (95% CI: -0.712 to -0.171). Details are given in Table 1.

**Table 1:** Baseline characteristics. Values are presented as mean ± SD. Group differences were analyzed using the chi² test to assess significance and Kendall’s tau-b to evaluate effect size.

Participants	Junior (n = 17, <3 years)	Senior (n = 25, ≥3 years)	p-value (effect size)
ICU experience (years), mean ± SD	1.59 ± 0.50	4.00 ± 0.60	p < 0.001 (0.796)
Self-perceived competence level, mean ± SD	3.65 ± 1.30	4.56 ± 1.00	p = 0.003 (0.439)

Accessing the overall usability of the ultrasound probe in VR

**Figure 1:**
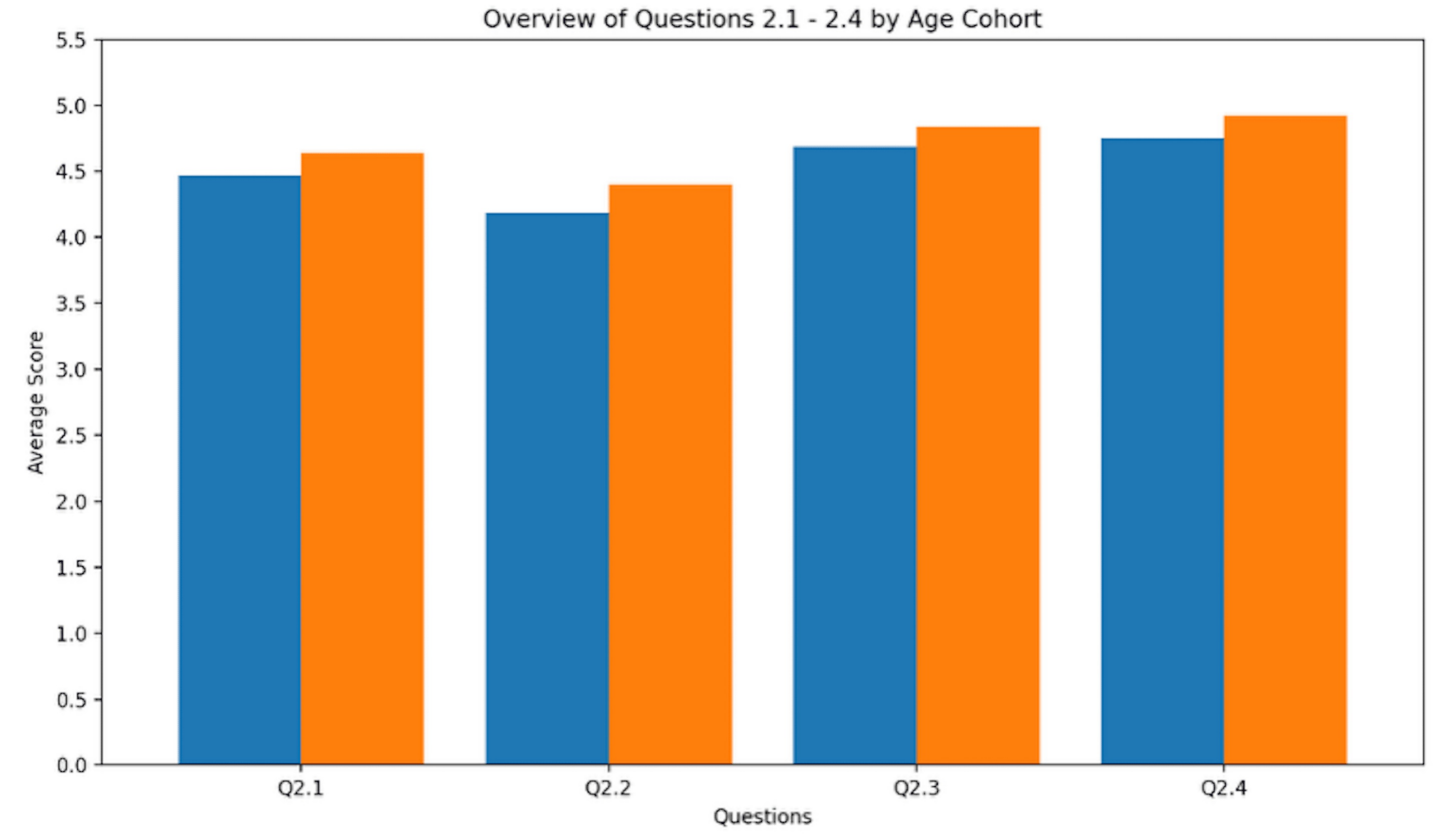
Usability features. Values for usability features are given as mean (SD). Q2.1 - Ultrasound probe usability in virtual environment: junior = 4.47 (0.62); senior = 4.64 (0.57). Q2.2 - Button and control arrangement intuitiveness: junior = 4.19 (0.91); senior = 4.40 (0.76). Q2.3 - Probe positioning capability in virtual environment: junior = 4.69 (0.48); senior = 4.84 (0.37). Q2.4 - Probe handling and operation comfort: junior = 4.75 (0.45); senior = 4.92 (0.28).

The user-friendliness was assessed, with a focus on operability in the VR environment. There was no significant difference between the junior and senior groups. The median level was 5, except for Q2.2, for which juniors had a median score of 4. The details are given in Figure 1; for better differentiation, the results are given as means.

Image quality in the VR environment

**Figure 2:**
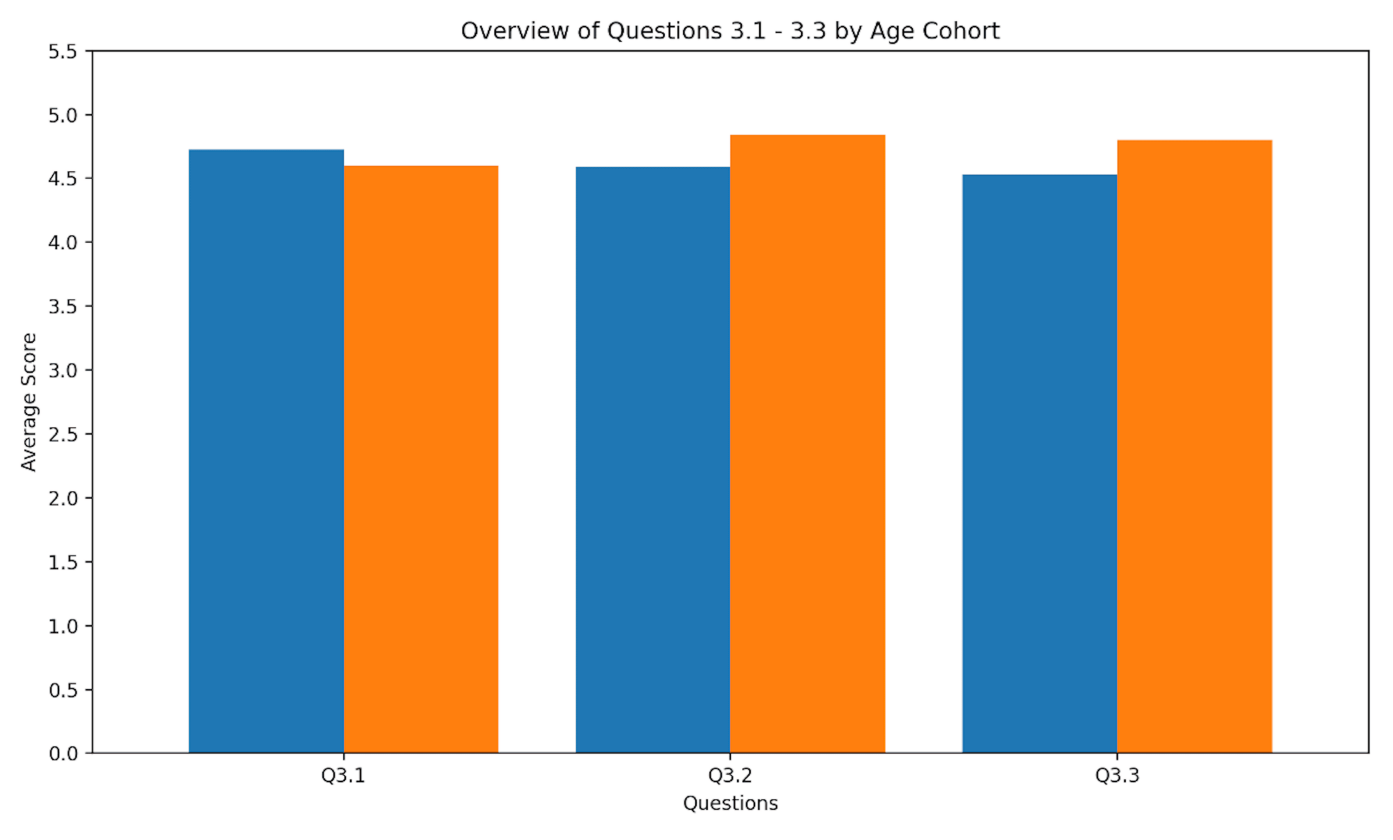
Image quality. Values for image quality are given as mean (SD). Q3.1 - Projection of ultrasound image based on anatomical landmarks: junior = 4.73 (0.47); senior = 4.60 (0.70). Q3.2 - Image quality in VR: junior = 4.59 (0.87); senior = 4.84 (0.47). Q3.3 - Interpretation of anatomical structures: junior = 4.53 (0.94); senior = 4.80 (0.50).

The image quality in the VR environment was assessed by asking about the feasibility of projecting the image at the right location, the image resolution, and the identifiability of anatomical structures. Additionally, we did not find differences between juniors and seniors; details are given in Figure 2. Overall, participants rated the image quality as excellent, with consistent scores across both junior and senior groups.

Subjective satisfaction and future implications

**Figure 3:**
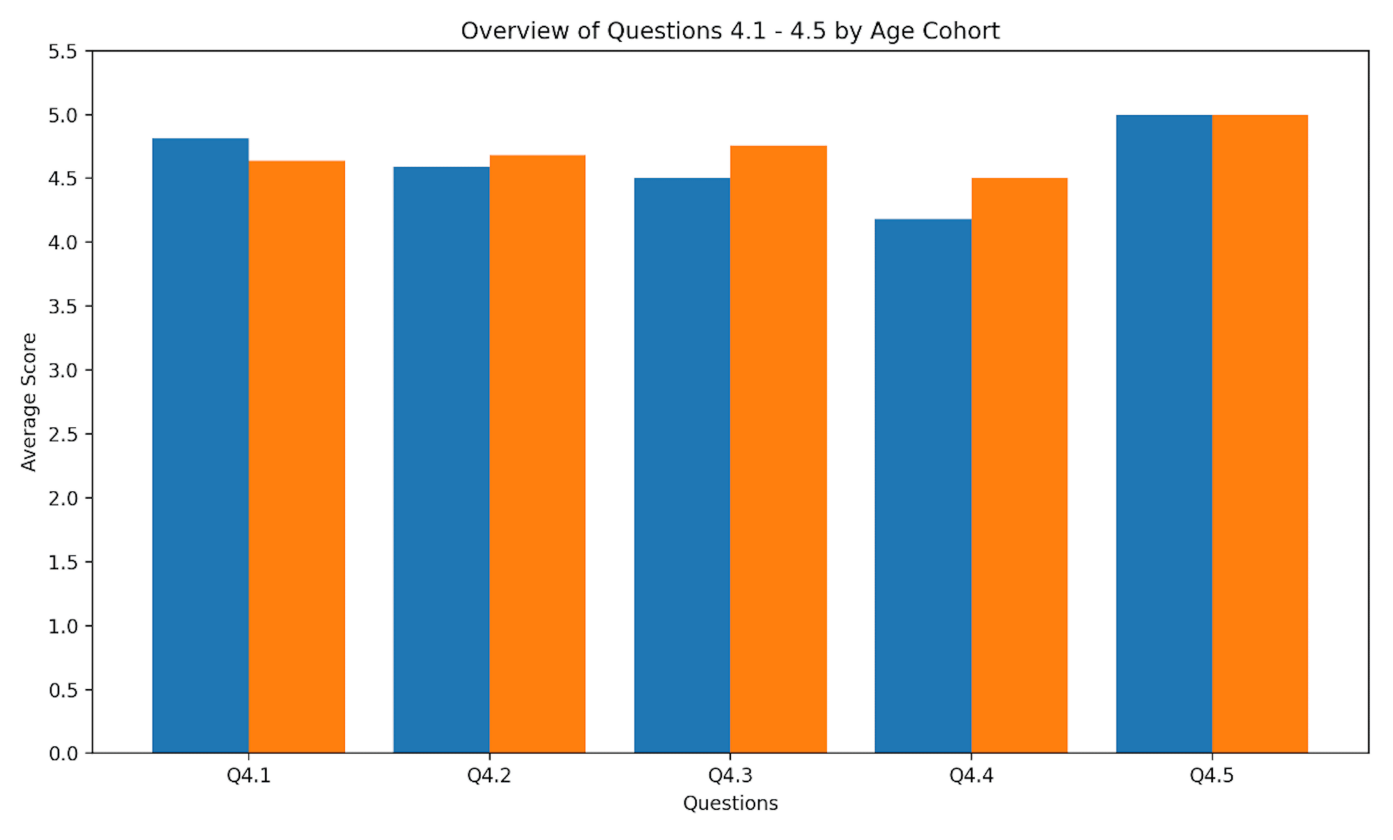
Satisfaction and future implications. Values for satisfaction and future implications are given as mean (SD). Q4.1 - Overall satisfaction with probe and virtual environment combination: junior = 4.81 (0.54); senior = 4.64 (0.49). Q4.2 - Potential contribution to patient safety through direct visualization: junior = 4.59 (0.71); senior = 4.68 (0.75). Q4.3 - Recommendation for future applications: junior = 4.50 (0.73); senior = 4.76 (0.52). Q4.4 - Efficiency improvement through combination with virtual procedure: junior = 4.18 (0.98); senior = 4.50 (0.71). Q4.5 - Potential contribution to education and training: junior = 5.00 (0.00); senior = 5.00 (0.00).

With five questions, we asked for future implications in terms of patient safety, efficiency, and education. Additionally, we did not find any significant differences with high median rates of 5, except for juniors regarding question Q4, for which the median was 4 (IQR: 1). Details are given in Figure 3.

Subjective rating

**Figure 4:**
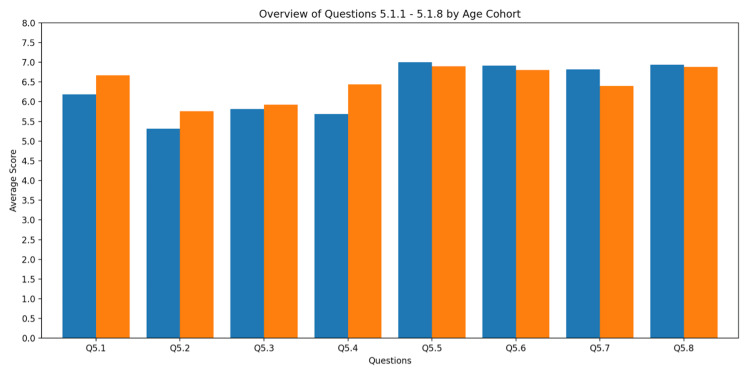
Subjective rating. Values for subjective rating are given as mean (SD). Q5.1 - Usability (hindering – supportive): junior = 6.19 (1.38); senior = 6.67 (0.56). Q5.2 - Usability (complicated – simple): junior = 5.31 (1.49); senior = 5.76 (1.27). Q5.3 - Efficiency (inefficient – efficient): junior = 5.81 (1.17); senior = 5.92 (1.12). Q5.4 - Clarity (confusing – clear): junior = 5.69 (1.70); senior = 6.44 (0.77). Q5.5 - Engagement (boring – exciting): junior = 7.00 (0.00); senior = 6.90 (0.32). Q5.6 - Interest (uninteresting – interesting): junior = 6.91 (0.30); senior = 6.80 (0.42). Q5.7 - Originality (conventional – original): junior = 6.82 (0.40); senior = 6.40 (0.52). Q5.8 - Novelty (traditional – innovative): junior = 6.94 (0.25); senior = 6.88 (0.33).

We tested the users’ perceived supportiveness, simplicity, efficiency, clarity, engagement, and innovativeness of the VR environment via a semantic differential on a seven-point Likert scale. High ratings were found for novelty but also for the supportiveness of the VR environment. Details are given in Figure 4.

Qualitative user feedback: Open questions and interviews

We collected in-depth qualitative feedback from participants through both open-ended survey questions and semi-structured interviews. The answers were categorized into positive aspects, challenges, and suggestions for improvement.

Positive Feedback

Image quality and visualization: Most of all, participants emphasized the realistic and well-positioned images (“perfect sight”, “very good illumination of the ultrasound image”, “The image is exactly where it needs to be”).

Usability and intuitive operation: The system was perceived as easy to use and intuitive (“intuitive sterile work”, “ease of use”).

Sterile work and space efficiency: Particularly in interviews, users appreciated how the system supports sterile procedures and efficient use of space (“allows sterile handling”, “no space wasted”).

Innovation and future orientation: Users expressed enthusiasm about the technology’s paradigm shifting potential (“An amazing experience. Definitely the future”).

Challenges and Limitations

Users encountered various difficulties during interaction with the system. The first category identified was “Image Clarity in the Near Field and Depth Perception”. A common concern in both sources was the limited resolution and lack of 3D perception in the near field (“See through a bit grainy”, “No 3D-view in the near field”).

System setup and interface: Initial unfamiliarity and complex setup were noted by some participants (“Need to get used to setup and interface”, “setup was difficult”).

Technical limitations: One Interviewee mentioned the limited performance of handheld probes compared to stationary systems.

Suggestions for Improvement

Related to the suggestions under the section "Challenges", the following categories were formulated: “Enhancement of Image Resolution”, particularly for tasks such as guide-wire handling in the near field (“Real-world images should be better”); “Improved Usability and Workflow Integration”, calls were made for a more streamlined, ready-to-use system (“Put-on and go solution”); and “System Integration”, suggestions included better integration of hardware components to enhance clinical workflow and ergonomics.

Observation

Twenty-two participants were observed. General orientation and setup proceeded smoothly, with minor delays in three cases due to difficulty finding the correct settings.

For specific observations, the procedure was divided into three parts. First, we observed the CVC setup preparation. Here, we did not see any problems. Puncture success was 100%, although three participants required a second attempt.

As a part of the procedure, guidewire handling posed some challenges, but these were not consistently observed. In two participants, threading the catheter over the guidewire took three attempts; in one participant, there were two attempts within a time frame of less than 5 seconds.

## Discussion

In this study, we present a novel proof-of-concept study evaluating virtual ultrasound-guided CVC insertion via the Apple Vision Pro and a wireless ultrasound probe (GE Vscan Air™ CL). To our knowledge, this is the first evaluation focused on the feasibility of this procedure. Previous studies included augmented reality procedures with see-through devices, which have shown remarkable results concerning first-attempt success and access-related complications [6-8].

The combination of immersive mixed reality with real-time procedural guidance represents a forward-thinking approach that addresses difficulties in using classic stand-alone ultrasound devices in terms of ergonomics, hand‒eye coordination, screen placement, and AR-related problems such as inadequate resolution, sensitivity to light conditions, and a non-adjustable field of view.

We were able to highlight several positive outcomes, including intuitive handling and high ratings for visualization, ergonomics, and immersion. The participants especially appreciated easy image positioning and the ability to maintain a sterile setup, which suggests strong potential for its practical use. Also, we could show that juniors and seniors did not have significant differences in most categories. Since both gave high ratings, this could suggest the system could be useful for experts and novices alike.

However, challenges were also identified, particularly blurriness and limited depth perception in the near field. Some users experienced minor latency and needed time to become familiar with the interface.

Suggestions for improvement focus on enhancing the system's optical resolution, particularly in the near field. The problem in this case concerns the near-field imaging performance of the AVP, which appears blurred in the 3D display at viewing distances of around 20-30 cm. Native integration with VisionOS is recommended to increase performance and streamline setup. Especially for individual setups, there should be a permanent guest mode to allow fast interchangeability between users. The introduction of other gesture controls could further support hands-free, sterile operations. Secondly, the setup for each participant was recognized as hindering everyday use. One solution could be to assign a dedicated device per user or implement a mechanism for saving user-specific setup configurations. Further updates to the operating system might also address this issue.

The strength of this study lies in the use of different methods to evaluate the new system (observation, questionnaire, observation, and interview). We combined quantitative (Likert scale) and qualitative (Mayring-based content analysis) methods. Hereby, we hope to obtain a deep understanding of the feasibility and implications for improvement.

This study has several limitations. First, it is of an explorative nature, to generate hypotheses, not to test them. While our findings show high usability and satisfaction in a simulated environment, clinical effectiveness in real patients remains to be validated.

Also, the sample size of 42 participants (in subgroup 22 participants) was relatively small. While adequate for an exploratory pilot study, this limits the generalizability of the findings. Secondly, the study was conducted in a simulated environment using mannequins. This setup may not fully reflect real-life clinical challenges such as venous filling status, soft tissue resistance, or real-time stressors.

The lack of a control group prevents direct comparison with traditional ultrasound-guided procedures. Therefore, our findings regarding improvements in ergonomics, accuracy, or safety should be interpreted as perceived rather than objectively validated, within a simulated environment. One methodological limitation might be that the observer-rated outcomes are rather subjective than measurable (e.g., guidewire difficulty). Other limitations are the absence of long-term follow-up to evaluate retention of training effects.

Future considerations

A logical next step would be real-world clinical testing in patients, allowing for comparison with standard ultrasound techniques. Additionally, extended feasibility testing across different procedures, such as arterial line placement and ECMO cannulation, and in various clinical contexts, including emergencies, might further help evaluate usability, especially considering patient safety. Another strong potential might be its use in education and residency training. Here, improving ergonomics and supervision by projecting the user’s view on a second observer screen might help make the CVC easier to learn and thus mitigate potential risks.

## Conclusions

This study provides promising early evidence supporting the use of mixed-reality devices like the Apple Vision Pro in procedural training and potentially in clinical practice. While technical enhancements are warranted, the overall user experience and methodological robustness suggest high potential for future applications, particularly in medical education and real-time procedural guidance. A randomized controlled trial comparing AVP-guided vs. standard ultrasound-guided CVC insertion in real patients could evaluate clinical benefit and safety.

## Appendices

**Table 2:** Usability questionnaire. This questionnaire evaluates users' experiences with an ultrasound probe in a virtual environment across three domains. Responses include fixed-choice Likert-type scales (1 = strongly disagree to 5 = strongly agree), bipolar semantic differential scales (1 to 7), and open-ended questions for qualitative feedback.

Section	Question number	Question text	Answer options
Demographic data	1.1	How many years of experience do you have in intensive care medicine?	0 - 1; 1 - 2; 2 - 3; 3 - 5; >5
Demographic data	1.2	I have a high level of competence in placing large-bore central venous catheters.	Strongly disagree; Rather disagree; Neutral; Rather agree; Strongly agree
Usability	2.1	The ultrasound probe was easy to operate in the virtual environment.	Strongly disagree; Rather disagree; Neutral; Rather agree; Strongly agree
Usability	2.2	The buttons and controls were intuitively arranged in the virtual environment.	Strongly disagree; Rather disagree; Neutral; Rather agree; Strongly agree
Usability	2.3	I was able to position the probe in the virtual environment without difficulty.	Strongly disagree; Rather disagree; Neutral; Rather agree; Strongly agree
Ergonomics	3.1	The ultrasound probe was comfortable to hold and operate in the virtual environment.	Strongly disagree; Rather disagree; Neutral; Rather agree; Strongly agree
Imaging comprehensibility	4.1	It was easy to project the ultrasound image onto the body using anatomical landmarks.	Strongly disagree; Rather disagree; Neutral; Rather agree; Strongly agree
Imaging comprehensibility	4.2	The quality of the ultrasound images in the VR environment was clear and comprehensible.	Strongly disagree; Rather disagree; Neutral; Rather agree; Strongly agree
Imaging comprehensibility	4.3	The representation of anatomical structures was easy to interpret.	Strongly disagree; Rather disagree; Neutral; Rather agree; Strongly agree
Imaging comprehensibility	4.4	I could identify the desired structures quickly and easily.	Strongly disagree; Rather disagree; Neutral; Rather agree; Strongly agree
Subjective satisfaction	5.1	Overall, I was very satisfied with the combination of the probe and the virtual environment.	Strongly disagree; Rather disagree; Neutral; Rather agree; Strongly agree
Subjective satisfaction	5.2	I have the impression that direct visualization can potentially contribute to patient safety.	Strongly disagree; Rather disagree; Neutral; Rather agree; Strongly agree
Subjective satisfaction	5.3	I would recommend this probe for future applications.	Strongly disagree; Rather disagree; Neutral; Rather agree; Strongly agree
Subjective satisfaction	5.4	I believe this probe makes working with the virtual method more efficient.	Strongly disagree; Rather disagree; Neutral; Rather agree; Strongly agree
Subjective assessment	6	I found the use of the probe in the virtual environment to be:	
Subjective assessment	6.1	Hindering — supportive	Scale rating (e.g., 1 to 7)
Subjective assessment	6.2	Complicated — simple	Scale rating (e.g., 1 to 7)
Subjective assessment	6.3	Inefficient — efficient	Scale rating (e.g., 1 to 7)
Subjective assessment	6.4	Confusing — clear	Scale rating (e.g., 1 to 7)
Subjective assessment	6.5	Boring — exciting	Scale rating (e.g., 1 to 7)
Subjective assessment	6.6	Uninteresting — interesting	Scale rating (e.g., 1 to 7)
Subjective assessment	6.7	Conventional — original	Scale rating (e.g., 1 to 7)
Subjective assessment	6.8	Traditional — innovative	Scale rating (e.g., 1 to 7)
Open questions	7.1	What did you particularly like?	Open text
Open questions	7.2	What difficulties or challenges did you encounter during use?	Open text
Open questions	7.3	Do you have suggestions for improvement?	Open text

**Table 3:** Observation items and interview. Free-text qualitative observer feedback across three phases: first impressions, task execution (e.g., CVC placement), and debriefing. Questions FI1–FI3 and TE1–TE3 refer to observations, while I1–I4 are interview questions. CVC: central venous catheter.

Section	Question number	Question text	Answer options
First impression	FI1	What does the test person see first? What do they do first?	Open text
First impression	FI2	What stands out positively?	Open text
First impression	FI3	What stands out negatively? Where are the problems at the beginning of the interaction?	Open text
Task execution	TE1	Observations on the preparation of the CVC application, setting up the image, etc.: What steps does the test person perform, what is noticeable, where are these obstacles?	Open text
Task execution	TE2	Observations on puncture and wire insertion/control. What steps does the test person perform, what is noticeable, and where are these obstacles?	Open text
Task execution	TE3	Observations on threading the CVC onto the wire. Which steps does the test person perform, what is noticeable, and where are these obstacles?	Open text
Interview	I1	General positive comments?	Open text
Interview	I2	Negative comments. What did the test person find annoying about the application or the interaction?	Open text
Interview	I3	What neutral comments did the test person make, e.g., to improve the application?	Open text
Interview	I4	Other observations and remarks:	Open text
